# Supplementary material for: Soybean Calmodulin-Binding Transcription Activators, GmCAMTA2 and GmCAMTA8, Coordinate the Circadian Regulation of Developmental Processes and Drought Stress Responses
Source: Int J Mol Sci. 2023 Jul 14;24(14):11477. doi: 10.3390/ijms241411477 (PMC10380932; doi:10.3390/ijms241411477)
Supplement: Supplementary file 1 [file ijms-24-11477-s001.zip › ijms-2440709-supplementary.pdf]

Supplementary Informations

Soybean Calmodulin-binding Transcription Activators, GmCAMTA2 and GmCAMTA8, Coordinate the Circadian Regulation of Developmental Processes and Drought Stress Responses

Dongwon Baek [1,†], Hyun Min Cho [2,†], Ye Jin Cha [2], Byung Jun Jin [3], Su Hyeon Lee [2], Mi Suk Park [1], Hyun Jin Chun [3,*], Min Chul Kim [1,2,3,*]

1 Plant Molecular Biology and Biotechnology Research Center, Gyeongsang National University, Jinju 52828, Republic of Korea; dw100@hanmail.net (D.B.); misugip@hanmail.net (M.S.P.)
2 Division of Applied Life Science (BK21 Four), Gyeongsang National University, Jinju 52828, Republic of Korea; hmcho86@gnu.ac.kr (H.M.C.); cdw3280@naver.com (Y.J.C.); leesuhyeon86@gmail.com (S.H.L.)
3 Institute of Agriculture and Life Science, Gyeongsang National University, Jinju 52828, Republic of Korea; scv5789@naver.com
* Correspondence: hj_chun@hanmail.net (H.J.C.); mckim@gnu.ac.kr (M.C.K.); Tel.: +82-55-772-1874 (H.J.C. & M.C.K)
† These authors contributed equally to this work.

Supplementary Materials:
Supplementary Figure S1. Diurnal expression of *GmCAMTA*s in the Diurnal web database.
Supplementary Figure S2. Transcriptional expression of *GmCAMTAs* in soybean tissues using qRT-PCR analysis.
Supplementary Figure S3. RT-PCR analysis in *Arabidopsis* transgenic plants overexpressing *GmCAMTA2* and *GmCAMTA8*.
Supplementary Table S1. List of 15 *GmCAMTA* genes in the Phytozome database.
Supplementary Table S2. List of primers in this study.

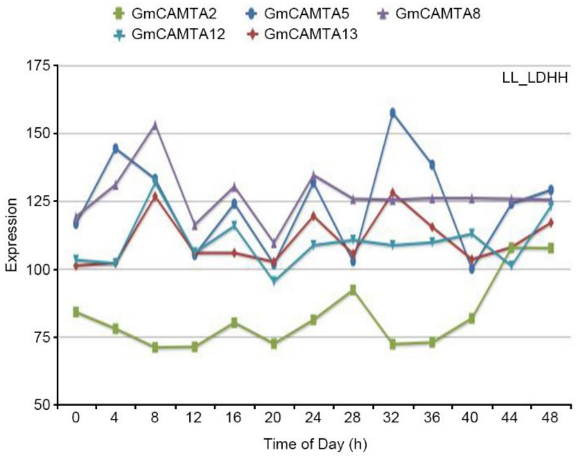

Supplementary Figure S1. Diurnal expression of *GmCAMTA*s in the Diurnal web database.

The diurnal expression of *GmCAMTA2*, *GmCAMTA5*, *GmCAMTA8*, *GmCAMTA12*, and *GmCAMTA13* under LL_LDHH condition using the Diurnal database tool (http://diurnal.mocklerlab.org/). The diurnal expression data of other *GmCAMTAs* were not available in the Diurnal web database. The "LL_LDHH" means a constant light for 24 h (LL), light 12h/dark 12h conditions (LD), and a constant hot temperature (31 °C; HH).

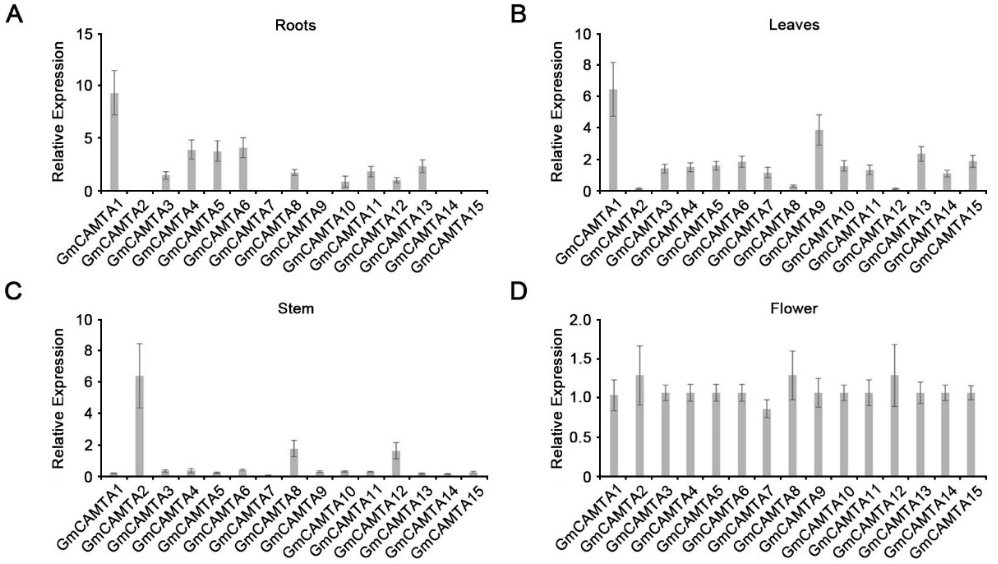

Supplementary Figure S2. Transcriptional expression of GmCAMTAs in soybean tissues using qRT-PCR analysis. The expression levels of *GmCAMTAs* were analyzed in different tissues of soybean plants using qRT-PCR. The total RNA was extracted from various tissues, including (A) roots, (B) leaves, (C) stem, and (D) flower, at the V4 stages of WT soybeans grown under long-day conditions (16h light / 8h dark). qRT-PCR analysis was performed with each *GmCAMTA*-specific primer (Supplementary Table S2). *GmTUBULIN* was used as an internal control for normalization. Error bars represent the SD of three biological replicates, each with three technical replicates.

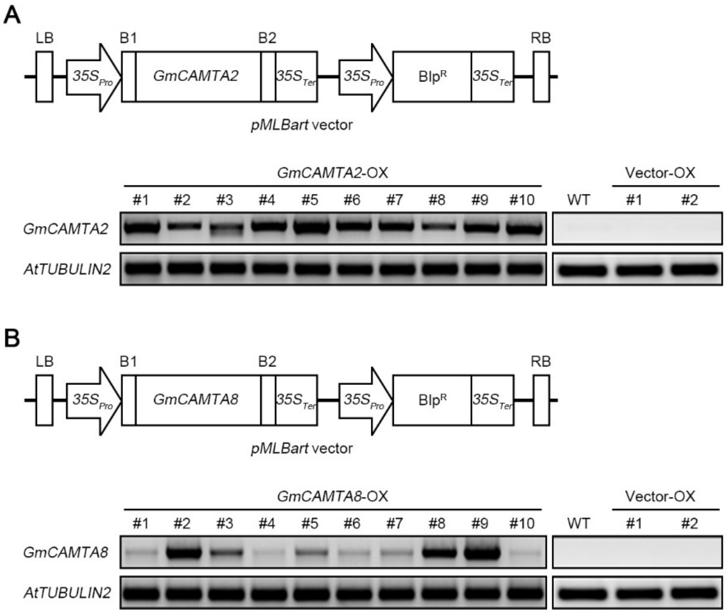

Supplementary Figure S3. RT-PCR analysis in *Arabidopsis* transgenic plants overexpressing *GmCAMTA2* and *GmCAMTA8*. Upper panels in (A) and (B); Schematic representation of the *pMLBart* binary vectors used for generating *Arabidopsis* transgenic plants overexpressing *GmCAMTA2* (A) and *GmCAMTA8* (B). Bottom panels in (A) and (B); Analysis of RT-PCR. Total RNAs isolated from each 10-day-old seedling of *Arabidopsis* T1 transgenic plants overexpressing *GmCAMTA2* (A) and *GmCAMTA8* (B). The analysis of RT-PCR was performed with each *GmCAMTAs*-specific primer (Supplementary Table S2). The *AtTUBULIN2* in *Arabidopsis* was used as an internal control.

Supplementary Table S1. List of 15 *GmCAMTA* genes in the Phytozome database.

Gene Name	Locus No.	Chromosome No.	Genome Length (bp)	Transcript Length (bp)	CDS Length (bp)	Protein Length (aa)
GmCAMTA1	*Glyma.05G 178200*	5	8,069	3,889	3,369	1,123
GmCAMTA2	*Glyma.08G 135200*	8	8,007	3,872	3,309	1,103
GmCAMTA3	*Glyma.15G 053600*	15	11,253	3,830	3,267	1,089
GmCAMTA4	*Glyma.08G 072100*	8	11,294	3,813	3,240	1,080
GmCAMTA5	*Glyma.05G 117000*	5	10,456	3,791	3,267	1,089
GmCAMTA6	*Glyma.08G 178900*	8	11,604	3,947	3,246	1,082
GmCAMTA7	*Glyma.17G 038800*	17	8,414	3,665	3,000	1,000
GmCAMTA8	*Glyma.15G 143400*	15	9,976	3,274	2,736	912
GmCAMTA9	*Glyma.09G 038300*	9	10,333	3,378	2,736	912
GmCAMTA10	*Glyma.05G 148300*	5	10,592	3,637	2,952	984
GmCAMTA11	*Glyma.18G 005100*	18	9,037	3,570	2,889	963
GmCAMTA12	*Glyma.17G 031900*	17	8,109	3,606	2,769	923
GmCAMTA13	*Glyma.07G 242000*	7	8,858	3,425	2,766	922
GmCAMTA14	*Glyma.11G 251900*	11	8,300	3,196	2,733	911
GmCAMTA15	*Glyma.08G 105200*	8	8,020	3,220	2,898	966

Supplementary Table S2. List of primers in this study.

Gene	Primer	Direction	Sequence (5’→3’)	Purpose
GmCAMTA1 (*Glyma.05G178200*)	MG-2943	Forward	GAAAAGGTAGTGGTTTGCGTG	Analysis of qRT-PCR or RT-PCR
	MG-2944	Reverse	CTTTGCTCTGTTTGCTTCCTG	
GmCAMTA2 (*Glyma.08G135200*)	MG-2931	Forward	AAGGCTGGAAGTGTGGATG	
	MG-2932	Reverse	TTGGTTCCCTTCACTTGTCG	
GmCAMTA3 (*Glyma.15G053600*)	MG-2957	Forward	AGGGACGGAAACAAAAGGAAG	
	MG-2958	Reverse	ACCCTTGTTAGATGCCTTGG	
GmCAMTA4 (*Glyma.08G072100*)	MG-2947	Forward	GTTCAGTATCCAGAGGCACG	
	MG-2948	Reverse	TCCCTTGGTTTGACGGAAG	

GmCAMTA5 (*Glyma.05G117000*)	MG-2963	Forward	GTTCAGTATCCAGAGGCACG	
	MG-2964	Reverse	CACCATCAACTGTCTCTTCCG	
GmCAMTA6 (*Glyma.08G178900*)	MG-2951	Forward	AGGGACGGAAACAAAAGGAAG	
	MG-2952	Reverse	AACCCTCGTTAGATGCCTTG	
GmCAMTA7 (*Glyma.17G038800*)	MG-2959	Forward	CAGATCCACCTGTTATGCCTC	
	MG-2960	Reverse	GCTTCTCTTACAGTCTTCCCATC	
GmCAMTA8 (*Glyma.15G143400*)	MG-2933	Forward	CTGCGTTGGCGTTTAAAGAG	
	MG-2934	Reverse	AATCACTTTCCTGCGTCTCC	
GmCAMTA9 (*Glyma.09G038300*)	MG-2953	Forward	GTAGTCCTTTATCTTCGGGTGG	
	MG-2954	Reverse	GTTCATCCATGTTCCAAAGCTG	
GmCAMTA10 (*Glyma.05G148300*)	MG-2941	Forward	TGACATCAATGGGTGGACTG	
	MG-2942	Reverse	GGTTGGATCTTGTGCATTTGG	
GmCAMTA11 (*Glyma.18G005100*)	MG-2961	Forward	TGGGCAGTTGGAATCTTGG	
	MG-2962	Reverse	CGGAACACGCTGAGAAAATC	
GmCAMTA12 (*Glyma.17G031900*)	MG-2935	Forward	CTCGCATACAGTGTACATACCG	
	MG-2936	Reverse	ACGACACCAACAGACCAAAG	
GmCAMTA13 (*Glyma.07G242000*)	MG-2945	Forward	TCTTTGGTCTGTTGGTGTGG	
	MG-2946	Reverse	ATCGACAGTTTTGACCTGGAG	
GmCAMTA14 (*Glyma.11G251900*)	MG-2955	Forward	ATTTCTCTGCCATGACTCGG	
	MG-2956	Reverse	ATGCCGTCCTGAATTATCTCTG	
GmCAMTA15 (*Glyma.08G105200*)	MG-2949	Forward	GTCTTTTCTCTGTCATCCCTCG	
	MG-2950	Reverse	TGCACAGAGTAACCTTTCCAG	
GmTUBULIN (*Glyma.17G258300*)	MG-2937	Forward	TGGCCGTTACCTGACAGCAT	
	MG-2938	Reverse	CTCGGAGGGATGTCACACAC	
AtRD29A (*AT5G52310*)	MG-2000	Forward	CCTGAAGTGATCGATGCACCAG	
	MG-2001	Reverse	TGGTGTAATCGGAAGACACGAC	
AtRD29B (*AT5G52300*)	MG-2002	Forward	GTGAAGATGACTATCTCGGTGG	
	MG-2003	Reverse	CACCACTGAGATAATCCGATCC	
AtP5CS2 (*AT3G55610*)	MG-2018	Forward	AGCAGCCTGTAATGCGATGG	
	MG-2019	Reverse	AAGTGACGCCTTTGGTTTGC	
AtKIN1 (*AT5G15960*)	MG-2016	Forward	CCAACAAGAATGCCTTCCAAGC	
	MG-2017	Reverse	GCTGCCGCATCCGATACACT	
AtTUBULIN2 (*AT5G62690*)	MG4028	Forward	TGGCATCAACTTTCATTGGA	
	MG-4029	Reverse	ATGTTGCTCTCCGCTTCTGT	
GmCAMTA2 (*Glyma.08G135200*)	MG-1523	Forward	CCATGGCTGAGGCCAGACTCTAC	Gene cloning
	MG-1281	Reverse	CTCAGCTGTGAAATTCTAAATCACCCC	
GmCAMTA8 (*Glyma.15G143400*)	MG-1530	Forward	CATCCCAAATATGGGGAAACCTTGTTC	
	MG-1531	Reverse	CATCCCAAATATGGGGAAACCTTGTTC	
